# Antimicrobial Resistance Genes in Bacteria Isolated From Japanese Honey, and Their Potential for Conferring Macrolide and Lincosamide Resistance in the American Foulbrood Pathogen *Paenibacillus larvae*

**DOI:** 10.3389/fmicb.2021.667096

**Published:** 2021-04-29

**Authors:** Mariko Okamoto, Masahiko Kumagai, Hiroyuki Kanamori, Daisuke Takamatsu

**Affiliations:** ^1^Division of Bacterial and Parasitic Disease, National Institute of Animal Health, National Agriculture and Food Research Organization, Tsukuba, Japan; ^2^Advanced Analysis Center, National Agriculture and Food Research Organization, Tsukuba, Japan; ^3^Institute of Crop Science, National Agriculture and Food Research Organization, Tsukuba, Japan; ^4^The United Graduate School of Veterinary Sciences, Gifu University, Gifu, Japan

**Keywords:** American foulbrood, antimicrobial susceptibility, bacteria in honey, macrolide and lincosamide resistance, mobile genetic elements, tylosin, *Paenibacillus larvae*

## Abstract

American foulbrood (AFB) is the most serious bacterial disease of honey bee brood. Spores of the causative agent *Paenibacillus larvae* are ingested by bee larvae via brood foods and germinated cells proliferate in the larval midgut. In Japan, a macrolide antibiotic, tylosin, is used as the approved prophylactic for AFB. Although tylosin-resistant *P. larvae* has yet to be found in Japan, it may emerge in the future through the acquisition of macrolide resistance genes from other bacteria, and bacteria latent in brood foods, such as honey, may serve as a source of resistance genes. In this study, to investigate macrolide resistance genes in honey, we attempted to isolate tylosin-resistant bacteria from 53 Japanese honey samples and obtained 209 isolates from 48 samples in the presence of 1 μg/ml of tylosin. All isolates were Gram-positive spore-forming bacteria mainly belonging to genera *Bacillus* and *Paenibacillus*, and 94.3% exhibited lower susceptibility to tylosin than Japanese *P. larvae* isolates. Genome analysis of 50 representative isolates revealed the presence of putative macrolide resistance genes in the isolates, and some of them were located on mobile genetic elements (MGEs). Among the genes on MGEs, *ermC* on the putative mobilizable plasmid pJ18TS1mac of *Oceanobacillus* strain J18TS1 conferred tylosin and lincomycin resistance to *P. larvae* after introducing the cloned gene using the expression vector. Moreover, pJ18TS1mac was retained in the *P. larvae* population for a long period even under non-selective conditions. This suggests that bacteria in honey is a source of genes for conferring tylosin resistance to *P. larvae*; therefore, monitoring of bacteria in honey may be helpful to predict the emergence of tylosin-resistant *P. larvae* and prevent the selection of resistant strains.

## Introduction

American foulbrood (AFB) is a highly contagious bacterial disease of honey bee brood and causes the collapse of honey bee colonies (Genersch, 2010). AFB is found in every area in which apiculture is practiced (Ellis and Munn, 2005). In Japan, AFB has also become widespread and the disease occurs every year throughout the country. As honey bees, in particular *Apis mellifera*, are the most important commercial pollinator contributing to the production of a variety of agricultural and horticultural crops, AFB is recognized as one of the most economically important diseases in apiculture worldwide.

The etiological agent of AFB is the Gram-positive, rod-shaped bacterium *Paenibacillus larvae*. Strains of this bacterium have been classified into five ERIC types (ERIC I–V) by repetitive-element PCR (Genersch et al., 2006; Beims et al., 2020) and 30 sequence types (STs) by multilocus sequence typing (MLST) (Morrissey et al., 2015)^1^. Strains with different genotypes differ in phenotypes, including virulence, at both the larval and colony levels (Genersch et al., 2005, 2006; Rauch et al., 2009; Beims et al., 2020). Among the five ERIC genotypes, ERIC I and II are the major types among the strains isolated from AFB cases. In Japan, ERIC I and II are the only genotypes found in isolates from AFB cases, and isolation of ERIC II strains has recently increased (Ueno et al., 2018). Similar to other *Paenibacillus* bacteria, *P. larvae* forms spores, which are the only infectious form of this organism (Genersch, 2010).

Honey bee larvae cannot get food by themselves and are fed by young worker bees called nurse bees. In a hive, worker larvae are fed worker jelly, a mixture of the hypopharyngeal and mandibular gland secretions of nurse bees, and then additionally fed some pollen and honey on or after the third day of larval life (Winston, 1987). Larvae are the most susceptible to *P. larvae* infection during the early larval stages, i.e., 12–36 h after egg hatching, and become infected through the ingestion of brood foods contaminated with *P. larvae* spores (Genersch, 2008). Ingested spores germinate and massively proliferate in the larval midgut. *P. larvae* vegetative cells then penetrate the peritrophic membrane, breach the midgut epithelium via the paracellular route, enter the haemocoel and kill the larvae (Genersch, 2008, 2010; Yue et al., 2008). Dead brood becomes ropy, dries out and then forms hard dark scales. The scales contain millions of *P. larvae* spores and drive disease transmission within and between colonies (Sturtevant, 1932; Bailey and Ball, 1991; Genersch, 2008). *P. larvae* spores may also exist in clinically healthy colonies. Indeed, analysis of honey samples harvested some years before the outbreak of AFB revealed that colonies were contaminated with spores several years before the detection of clinical symptoms (von der Ohe, 2003).

*P. larvae* spores are highly resistant to heat and chemical agents and can survive for many years in dried larval scales, hive products and equipment (OIE, 2018); therefore, burning of diseased colonies and contaminated hive materials is considered to be the most effective control measure for AFB. In several countries, antimicrobials have also been used as an alternative control measure. Oxytetracycline (OTC) is the most commonly used antimicrobial in the control of AFB (Reybroeck et al., 2012). However, OTC- and tetracycline-resistant *P. larvae* strains have been detected in several countries, including the United States, Canada and Argentina (Alippi, 2000; Miyagi et al., 2000; Evans, 2003; Krongdang et al., 2017). In previous studies, approximately 40% of Argentinian and 16% North American *P. larvae* isolates tested were OTC resistant (Alippi, 2000; Krongdang et al., 2017). The United States Food and Drug Administration additionally approved a macrolide antibiotic (tylosin tartrate [TS]) in 2005 and a lincosamide antibiotic (lincomycin hydrochloride [LCM]) in 2012 for the control of this disease; however, in the United States, TS- and/or LCM-resistant *P. larvae* were isolated from AFB cases in 2007, 2010 and 2013 (Krongdang et al., 2017).

OTC-resistant phenotypes observed in *P. larvae* develop, at least in part, due to plasmids carrying the tetracycline resistance gene [*tet*(L)], and the plasmids may be transferable across bacterial species (Murray and Aronstein, 2006; Alippi et al., 2007, 2014; Murray et al., 2007). In honey, many bacteria, including *Bacillus* and *Paenibacillus* species, are known to exist (Snowdon and Cliver, 1996; Alippi et al., 2004; Sinacori et al., 2014; Floyd et al., 2020). In a previous study, López et al. (2008) isolated *Bacillus cereus* strains carrying a variety of tetracycline and OTC resistance genes, including *tet*(L) and *tet*(K), from honey, and suggested that bacteria in honey are a reservoir for tetracycline resistance genes. In addition, the genome sequence analysis by Abrahamovich et al. (2018) revealed that *Bacillus thuringiensis* strain m401, a tetracycline-resistant isolate recovered from honey in Argentina, possessed a tetracycline resistance gene with high homology to *tet45*. As honey is a brood food for worker larvae, such antimicrobial-resistant bacteria in honey may contact *P. larvae* in the larval gut, and *P. larvae* may have acquired the tetracycline resistance genes from the honey-derived bacteria in the larval gut and become resistant to OTC.

In Japan, diseased colonies and contaminated hive materials have to be burned when AFB occurs; therefore, antimicrobials cannot be used for the treatment of AFB. However, to prevent AFB, 16-membered ring macrolides (mirosamicin [MRM] and TS) have been used in honey bee colonies for more than 20 years (Ueno et al., 2018). MRM was approved in 1999 by the Ministry of Agriculture, Forestry and Fisheries of Japan, and TS was additionally approved in 2017. As the sale of the MRM-containing prophylactic drug (Apiten for Honey bees; FEED ONE, Yokohama, Japan) was discontinued, TS is the only approved prophylactic available for controlling AFB at present. TS is currently used by dusting methods in Japan, and 150 mg of TS is used for 10,000 adult bees; i.e., the mixture consisting of 5 g of confectioner’s sugar and 50 mg of TS is sprinkled over the hive’s top bars three times at one-week intervals, and the dose can be scaled up depending on the number of adult bees. Although macrolide prophylactics have been used for a long time, macrolide-resistant *P. larvae* has yet to be found in Japan (Ueno et al., 2018). However, as mentioned above, TS-resistant *P. larvae* strains have been detected in North America (Krongdang et al., 2017). Although TS-resistant mechanisms of the North American *P. larvae* strains are unknown, we cannot exclude the possibility that TS-resistant *P. larvae* will emerge in Japan in the future through the acquisition of macrolide resistance genes, and the only approved prophylactic for AFB may therefore lose its effectiveness. As implied in the cases of OTC-resistant *P. larvae*, bacteria latent in honey may serve as a reservoir of antimicrobial resistance genes and *P. larvae* may acquire macrolide resistance genes from them in the larval gut. As best as we know, there is no information regarding the contamination of macrolide-resistant bacteria in honey. Therefore, we investigated the presence of TS-resistant bacteria in commercially available Japanese honey in this study. We also searched for macrolide resistance genes on mobile genetic elements (MGEs) by whole genome sequencing of representative TS-resistant honey-derived isolates and evaluated their potential for conferring TS resistance to *P. larvae*.

## Materials and Methods

### Japanese Honey Used in This Study

The 53 Japanese honey samples used in this study are listed in Supplementary Table 1 in Supplementary Materials. All of them were commercially available honey from different origins (i.e., different apiaries, different producers and/or different floral origins) and purchased between 2017 and 2018. Four honey samples (J12, J15, J24 and J42) were produced by Japanese honey bees, *Apis cerana japonica*, and the others were produced by European honey bees, *A. mellifera*. All samples were stored at room temperature until use.

### Isolation and Identification of Bacteria From Japanese Honey

#### Isolation of Bacteria From Honey Samples

For isolation of bacteria from honey, 20 ml of each honey sample was diluted to 50% (v/v) with sterile water and centrifuged at 11,000 rpm for 45 min. The supernatant was removed and the sediment was suspended with 1 ml of sterile water. One hundred microliters of each suspension was plated on Columbia blood agar (Becton, Dickinson and Company, Franklin Lakes, NJ, United States) containing 5% sheep blood (CSA), KSBHI agar (Arai et al., 2012), Lactobacilli MRS agar (Becton, Dickinson and Company) and modified GAM agar (Nissui Pharmaceutical Co., Ltd., Tokyo, Japan) containing 5% sheep blood (GAMSA). For selective isolation of TS-resistant bacteria, all agar media used for the isolation were supplemented with 1 μg/ml of TS. CSA was incubated at 35°C under air plus 5% CO_2_ conditions for two days, and the other agar media were incubated at 35°C under anaerobic conditions for four days. Colonies grown on the agar media were subcultured for purification. Pure-cultured bacterial isolates were suspended in LB broth (Becton, Dickinson and Company) containing 30% glycerol and stored at −80°C until use.

#### Identification of Honey-Derived Bacteria

To identify bacterial species of the isolates from Japanese honey, genomic DNA was extracted using InstaGene Matrix (Bio-Rad Laboratories, Inc., Hercules, CA, United States) and 16S rRNA gene sequences of the isolates were determined as described previously (Arai et al., 2012). Briefly, an approximately 1.5-kbp region of the 16S rRNA gene was amplified from the genomic DNA by TaKaRa ExTaq DNA polymerase (Takara Bio, Kusatsu, Japan) using primers F1 (5′-GAGTTTGATCCTGGCTCAG-3′) and R13 (5′-AGAAAGGAGGTGATCCAGCC-3′) (Dorsch and Stackebrandt, 1992). The amplified fragments were purified using QIAquick PCR purification kit (QIAGEN, Hilden, Germany) and sequenced by a BigDye terminator v3.1 cycle sequencing kit using a 3130*xl* Genetic Analyzer (Applied Biosystems, Tokyo, Japan). Sequencher ver. 5.4.6 (Gene Codes Corp., Ann Arbor, MI, United States) was used to assemble the sequences. The species or genus of the isolates was identified by analyzing the 16S rRNA gene sequences using the EzBioCloud server^2^ (Yoon et al., 2017). As 98.65% was proposed as the threshold of 16S rRNA gene sequence similarity for differentiating two species (Kim et al., 2014), when type strains with 98.65% or higher sequence similarity were not found by EzBioCloud, identification of the isolates was carried out only on a genus level. In addition, when more than one closest type strains were found by EzBioCloud, the isolates were also not identified on a species level but instead on a genus level. The 16S rRNA gene sequences determined in this study were deposited in the DNA Data Bank of Japan (DDBJ)/GenBank/European Molecular Biology Laboratory (EMBL) database under the accession numbers LC588427–LC588635. The presence of the cereulide synthetase gene was investigated in some *Bacillus* isolates using the *Bacillus cereus* (CRS gene) PCR Detection Kit (Takara Bio) according to the manufacturer’s instructions.

### Antimicrobial Susceptibility Tests for Bacterial Isolates From Japanese Honey

Two hundred and nine bacterial isolates derived from Japanese honey were cultured under the appropriate culture conditions listed in Supplementary Table 2 in Supplementary Materials and used for antimicrobial susceptibility tests. TS (Combi-Blocks Inc., San Diego, CA, United States), OTC (FUJIFILM Wako Pure Chemical Corp., Osaka, Japan) and LCM (Sigma-Aldrich, St. Louis, MO, United States) were used in this study. Except for the *Clostridium beijerinckii* isolate, minimum inhibitory concentrations (MICs) of these antimicrobials were determined by broth microdilution methods according to standard methods of Clinical and Laboratory Standards Institute (CLSI, M07-A10). Cation adjusted Mueller Hinton (MH) II broth (Becton, Dickinson and Company) was used for the tests and MICs were measured after a 24-h incubation at 35°C under aerobic conditions. For the quality control of each test, *Staphylococcus aureus* ATCC 29213, *Escherichia coli* ATCC 25922 and *Pseudomonas aeruginosa* ATCC 27853 cultured on MH agar (Becton, Dickinson and Company) for 20 h at 37°C under aerobic conditions were also employed. MICs for *C. beijerinckii* J33TS5 were measured by agar dilution methods according to CLSI, M07-A10, except that MICs were measured on GAMSA after a 24-h incubation at 35°C under anaerobic conditions. The antimicrobial concentration range employed for the tests was 0.25–256 μg/ml.

### Search for Antimicrobial Resistance Genes in Honey-Derived Bacteria by Genome Sequence Analysis

#### Genomic DNA Extraction

Bacterial cells cultured on agar media under appropriate culture conditions (Supplementary Table 2) were harvested and suspended in 550 μl of TE (10 mM Tris–HCl [pH 8.0] and 1 mM EDTA [pH 8.0]). The bacterial suspensions were then mixed with 60–80 μl of a mixed solution of two enzymes (100 mg/ml of lysozyme [Sigma-Aldrich] and 500 U/ml of mutanolysin [Sigma-Aldrich]) and incubated for 1 h at 37°C. After incubation, bacterial cells were lysed by adding 60–70 μl of 10% sodium dodecyl sulfate, and the lysates were extracted with an equal volume of phenol, phenol-chloroform-isoamyl alcohol (25:24:1) (PCI) and chloroform at least once, three times and once, respectively. Nucleic acids were then precipitated by ethanol, rinsed with 70% ethanol and dissolved in sterile H_2_O. DNA was further treated with 100 μg/ml of RNase (NIPPON GENE Co., Ltd., Tokyo, Japan) at 37°C for 2 h, extracted with PCI and chloroform twice and once, respectively, and precipitated by ethanol. Extracted DNA was rinsed with 70% ethanol, dissolved in 10 mM Tris–HCl (pH 8.5) and stored at −20°C until use.

#### Genome Sequencing

Among the 209 isolates from Japanese honey, 50 were selected for whole genome shotgun sequencing in this study (Table 1). Genome sequencing data were obtained using the Miseq and/or Novaseq 6000 platforms (Illumina, Inc., San Diego, CA, United States). Sequencing by the Miseq platform (300-bp paired-end reads) was performed at the Institute of Crop Science, National Agriculture and Food Research Organization as recommended by the manufacturer. Genomic libraries for the Miseq sequencing were prepared by the TruSeq Nano DNA low-throughput library preparation kit (Illumina). Sequencing by the Novaseq 6000 platform (151-bp paired-end reads) was performed by Macrogen Japan Corp (Tokyo, Japan). Genomic libraries for the Novaseq 6000 sequencing were prepared by the TruSeq DNA PCR Free kit (Illumina). Adapter sequences and low-quality bases in Illumina paired end reads were removed using Trimmomatic v.0.36 (Bolger et al., 2014) with options of ‘ILLUMINACLIP:TruSeq3-PE.fa:3:40:15 LEADING:20 TRAILING:20 MINLEN:20’. Whole genome *de novo* assembly was conducted using SPAdes v.3.13.2 with options of –careful and –cov-cutoff auto (Nurk et al., 2013). Genomes were annotated using Prokka v.1.14.5 (Seemann, 2014). The genome sequences were deposited in the DDBJ/GenBank/EMBL database under the DRA accession number DRA011480, BioProject accession number PRJDB11087 and BioSample accession numbers SAMD00277588–SAMD00277637.

**TABLE 1:** Bacterial isolates used for genome sequencing and macrolide resistance genes detected by Resistance Gene Identifier (https://card.mcmaster.ca/analyze/rgi).

Isolates	Species	MIC (μg/ml)^a^			Number of detected macrolide resistance gene			Macrolide resistance genes carried by MGEs^b^
		TS	LCM	OTC	Total	Criteria		
						Perfect or strict	Loose	
J1TS1	*Alkalihalobacillus clausii*	64	> 256	4	2	2	0	-
J1TS3	*Bacillus fordii*	8	128	8	4	1	3	-
J1TS5	*Paenibacillus macerans*	16	> 256	4	6	0	6	-
J2TS4	*Paenibacillus* sp.	8	> 256	8	4	1	3	-
J2TS5	*Bacillus licheniformis*	2	> 256	0.5	2	1	1	-
J2TS6	*Paenibacillus albilobatus*	4	256	32	2	0	2	-
J5TS1	*Bacillus licheniformis*	2	> 256	0.5	2	1	1	-
J5TS2	*Brevibacillus halotolerans*	8	32	8	2	0	2	-
J5TS4	*Bacillus* sp.	1	128	≤ 0.25	2	2	0	-
J6TS1	*Bacillus terrae*	> 256	128	8	5	1	4	-
J6TS2	*Bacillus sporothermodurans*	256	> 256	8	3	1	2	-
J6TS7	*Paenibacillus dendritiformis*	0.5	32	1	1	0	1	-
J8TS2	*Bacillus ruris*	> 256	>256	≤ 0.25	2	0	2	-
J11TS1	*Oceanobacillus* sp.	4	16	0.5	5	0	5	-
J14TS2	*Bacillus* sp.	8	16	2	5	0	5	-
J14TS5	*Paenibacillus lautus*	8	256	16	4	2	2	-
J15TS10	*Paenibacillus woosongensis*	8	128	≤ 0.25	2	0	2	-
J18TS1	*Oceanobacillus oncorhynchi* subsp. *incaldanensis*	> 256	>256	64	4	1	3	*ermC* in plasmid pJ18TS1mac (accession number: LC586958)
J19TS1	*Bacillus oleronius*	16	128	8	3	0	3	-
J19TS2	*Cohnella xylanilytica*	8	> 256	1	7	0	7	-
J21TS3	*Paenibacillus cookii*	64	> 256	8	5	0	5	-
J21TS7	*Paenibacillus cineris*	32	> 256	32	5	0	5	-
J22TS1	*Bacillus terrae*	8	64	16	5	1	4	-
J22TS3	*Paenibacillus* sp.	8	> 256	32	4	0	4	-
J23TS8	*Bacillus paralicheniformis*	4	> 256	16	2	1	1	-
J23TS9	*Paenibacillus* sp.	4	128	4	4	0	4	-
J25TS1	*Bacillus paralicheniformis*	2	> 256	16	2	1	1	-
J25TS5	*Paenibacillus faecis*	16	64	8	2	0	2	-
J26TS2	*Alkalihalobacillus clausii*	128	> 256	8	2	2	0	-
J27TS7	*Paenibacillus dendritiformis*	4	64	64	2	0	2	-
J27TS8	*Bacillus siralis*	32	> 256	≤ 0.25	2	0	2	-
J28TS4	*Paenibacillus lautus*	16	256	4	5	2	3	-
J31TS2	*Bacillus licheniformis*	4	> 256	0.5	2	1	1	-
J31TS3	*Paenibacillus lactis*	32	256	1	5	1	4	-
J31TS4	*Paenibacillus* sp.	8	256	≤ 0.25	2	0	2	-
J31TS6	*Brevibacillus reuszeri*	8	> 256	8	2	1	1	-
J32TS2	*Alkalihalobacillus clausii*	> 256	>256	4	2	2	0	-
J32TS6	*Virgibacillus pantothenticus*	256	> 256	1	3	0	3	-
J34TS1	*Paenibacillus azoreducens*	2	32	64	2	0	2	-
J36TS2	*Bacillus paralicheniformis*	2	256	8	2	1	1	-
J40TS1	*Paenibacillus montaniterrae*	4	> 256	8	3	0	3	-
J41TS2	*Bacillus sonorensis*	2	128	8	3	0	3	-
J41TS4	*Paenibacillus apis*	4	64	1	3	0	3	-
J41TS8	*Bacillus* sp.	2	> 256	8	2	1	1	-
J41TS12	*Paenibacillus antibioticophila*	8	64	0.5	3	0	3	-
J42TS3	*Paenibacillus vini*	32	32	1	2	0	2	-
J43TS3	*Ornithinibacillus bavariensis*	1	128	1	3	0	3	-
J43TS9	*Paenibacillus cineris*	64	> 256	64	4	0	4	*lsaB* and *oleC* in ICE
J45TS6	*Paenibacillus* sp.	32	> 256	64	2	1	1	*ermL* and *ermB* in plasmid pJ45TS6 (accession number: LC597664)
J53TS2	*Paenibacillus* sp.	8	32	0.5	4	0	4	-

*^a^MIC, minimum inhibitory concentration; TS, tylosin; LCM, lincomycin; OTC, oxytetracycline. ^b^MGEs, mobile genetic elements. ICE, integrative conjugative element. MGEs were identified by PlasmidFinder 2.1 (https://cge.cbs.dtu.dk//services/PlasmidFinder/), PHAST (http://phast.wishartlab.com) and ICEberg 2.0 (http://db-mml.sjtu.edu.cn/ICEberg/). Intact prophage was not detected.*

#### Screening of Antimicrobial Resistance Genes and MGEs From Genome Sequence Data

Putative antimicrobial resistance genes in the genome sequences were searched using Resistance Gene Identifier (RGI) in the Comprehensive Antibiotic Resistance Database (CARD)^3^ (McArthur et al., 2013). In this study, genes that exhibited > 50% best identity (percent identity of match to top hit in CARD) and percentage length of 60–140% with the reference sequence (for further details, see^4^) were selected as antimicrobial resistance genes. Integrative conjugative elements (ICEs) and prophages were searched using ICEberg 2.0^5^ (Liu et al., 2019) and PHAST^6^ (Zhou et al., 2011), respectively. Scaffolds including plasmid replicons were selected by PlasmidFinder 2.1^7^ (Carattoli et al., 2014), and complete sequences of the plasmids were determined by PCR and primer walking using the primers and conditions listed in Supplementary Table 3 in Supplementary Materials. The plasmid sequences were deposited in the DDBJ/GenBank/EMBL database under the accession numbers LC586958 (pJ18TS1mac), LC596398 (pJ18TS1tet) and LC597664 (pJ45TS6).

### Construction of Putative Macrolide Resistance Gene Expression Vectors and Introduction of the Vectors Into *P. larvae*

To construct putative macrolide resistance gene expression vectors, the *ermC* region in plasmid pJ18TS1mac from isolate J18TS1, the *ermL-ermB* and *ermB* regions in plasmid pJ45TS6 from isolate J45TS6, and the *lsaB* and *oleC* regions carried by a putative ICE in the isolate J43TS9 chromosome were amplified from the genomic DNA of the respective isolates by PCR using the primers and enzymes listed in Supplementary Table 3. The primers were designed to produce amplicons that contained the antimicrobial resistance gene and its Shine-Dalgarno sequence and/or putative promoter region. In this study, pMX2-TA, which was originally developed as a stable gene expression vector for the European foulbrood pathogen, *Melissococcus plutonius*, and contains the Gram-positive bacterial promoter sequence upstream of the multiple cloning sites (Takamatsu et al., 2020), was used. The PCR products were cloned into this plasmid vector via *Eco*RI and *Pst*I or *Bam*HI and *Pst*I sites in *E. coli* MC1061 (Casadaban and Cohen, 1980). *E. coli* was cultured in LB (Becton, Dickinson and Company) agar at 37°C under aerobic conditions, and chloramphenicol (CP) was added to the media at 16 μg/ml for maintenance of the expression vectors. Accuracy of the cloned sequences was confirmed by PCR and sequencing analyses. The resultant plasmids were then introduced into an ERIC II *P. larvae* strain, DTK384 (strain N in Hirai et al., 2016), by electroporation according to the procedures described by de Graaf et al. (2013) except that electrotransformed *P. larvae* cells were incubated in MYPGP broth at 37°C for 2 h 30 min–7 h 30 min without shaking, and transformants were selected on MYPGP agar supplemented with 16 μg/ml of CP. As a control, DTK384 possessing only pMX2-TA (DKT384control) was also constructed by introducing pMX2-TA into the strain. Resultant *P. larvae* strains are listed in Table 2.

**TABLE 2:** *Paenibacillus larvae* strains used in this study and their antimicrobial susceptibility.

Strain	Description^a^	Introduced macrolide resistance genes^b^	Putative function of the introduced resistance genes^b^	References	MIC (μg/ml)^c^		
					TS	EM	LCM
DTK384^*d*^	The genotype ERIC II-ST10 strain isolated from a diseased *Apis mellifera* larva in Japan	NA	NA	Hirai et al., 2016	0.25	0.125	0.25
DTK384ermC	The *ermC* expression vector-introduced DTK384	*ermC*	23S rRNA methyltransferase	This study	8	> 256	>256
DTK384ermLB	The *ermL-ermB* expression vector-introduced DTK384	*ermL* and *ermB*	23S rRNA methylase leader peptide (*ermL*), 23S rRNA methyltransferase (*ermB*)	This study	1	> 256	>256
DTK384ermB	The *ermB* expression vector-introduced DTK384	*ermB*	23S rRNA methyltransferase	This study	0.25	64	> 256
DTK384lsaB	The *lsaB* expression vector-introduced DTK384	*lsaB*	ATP-binding cassette F type ribosomal protection protein	This study	0.25	0.125	0.25
DTK384pJ18TS1mac	pJ18TS1mac-introduced DTK384	pJ18TS1mac with *ermC*	23S rRNA methyltransferase	This study	16	> 256	>256
DTK384control	pMX2-TA-introduced DTK384	NA	NA	This study	0.25	0.0625	0.25

*^a^ERIC, enterobacterial repetitive intergenic consensus; ST, sequence type. ^b^NA, not applicable. ^c^MIC, minimum inhibitory concentration; TS, tylosin; EM, erythromycin; LCM, lincomycin. ^d^DTK384 was named strain N in the previous report (Hirai et al., 2016).*

### RNA Extraction From *P. larvae* DTK384 and Its Derivatives, and Confirmation of Introduced-Antimicrobial Resistance Gene Expression by Reverse Transcription PCR (RT-PCR)

Total RNA of *P. larvae* was extracted from bacterial cells cultured on MYPGP agar (de Graaf et al., 2013) containing 16 μg/ml of CP at 35°C for two days under air plus 5% CO_2_ conditions using the RNeasy Mini kit supplemented with RNAprotect Bacteria Reagent and RNase-Free DNase Set, as recommended by the manufacturer (QIAGEN) with the following modifications: harvested and RNAprotect Bacteria Reagent-treated *P. larvae* cells were incubated in TE containing 10 mg/ml of lysozyme (Sigma-Aldrich) and 50 U/ml of mutanolysin (Sigma-Aldrich) at 37°C for 20 min and then lysed by β-mercaptoethanol-supplemented Buffer RLT in the RNeasy Mini kit. Extracted RNA was transcribed to single-strand cDNA using the PrimeScript RT reagent Kit with gDNA Eraser (Takara Bio). Expression of the introduced-antimicrobial resistance gene was confirmed by PCR using cDNA made from 10 ng of total RNA. To confirm the removal of DNA, the reverse transcriptase-untreated RNA was used as a template. All primers, polymerase and PCR conditions are listed in Supplementary Table 3.

### Filter Mating Test

In this study, isolates J18TS1 and J45TS6, which possess macrolide resistance plasmids, were used as donors, and MICs of mitomycin C (FUJIFILM Wako Pure Chemical Corp.) for the isolates were measured by broth microdilution methods using MYPGP broth as described above. As the recipient, rifampicin (RIF)-resistant *P. larvae* was used. To prepare the recipient strain, *P. larvae* DTK384 was cultured on MYPGP agar containing 2 μg/ml of RIF (FUJIFILM Wako Pure Chemical Corp.), and a spontaneously generated RIF-resistant strain was isolated and named strain M20. The MIC of RIF for this strain measured by agar dilution methods was 128 μg/ml. For mating tests, J18TS1 and J45TS6 cultured on MYPGP agar with 1 μg/ml of TS at 35°C for approximately 24 h under aerobic conditions and M20 cultured on MYPGP agar with 4 μg/ml RIF at 35°C for 48 h under air plus 5% CO_2_ conditions were separately inoculated into MYPGP broth and cultured at 35°C with shaking (200 rpm). For donors, TS was added to the broth at a final concentration of 1 μg/ml. When the optical density of the cultures at 600 nm reached 0.4–0.6, donor cells from 5 ml of the culture were further incubated in fresh MYPGP broth (no mitomycin C), and that containing 1/2 and 1/4 MIC of mitomycin C. After incubation at 35°C for 1 h, the donor cells were collected by centrifugation (11,000 rpm 10 min), washed three times with sterile saline to remove mitomycin C and suspended in 5 ml of fresh MYPGP broth. The donor and recipient cultures were then mixed at a ratio of 1:1, and the mixture was filtered through a 0.45-μm nitrocellulose filter (25 mm diameter). The filters were placed on MYPGP agar and incubated at 35°C for 24 h and 48 h under air plus 5% CO_2_ conditions. To select recipient cells that acquired macrolide resistant plasmids, the donor and recipient cells on the cultured filters were suspended in 1 ml of sterile saline. One hundred microliters of the suspension was placed on MYPGP agar containing both RIF (4 μg/ml) and TS (1 μg/ml), and incubated at 35°C for 48 h under air plus 5% CO_2_ conditions.

### Plasmid Extraction From J18TS1 and Introduction of pJ18TS1mac Into *P. larvae*

The plasmid pJ18TS1mac harbored by isolate J18TS1 was extracted using the illustra plasmidPrep Mini Spin Kit (GE Healthcare, Buckinghamshire, United Kingdom) according to the manufacturer’s instructions with the following modifications. Bacteria suspended in lysis buffer type 7 were treated with 0.2–1 mg/ml of lysozyme (Sigma-Aldrich) for 3–4 min at room temperature and then lysed by adding lysis buffer type 8. Extracted plasmids were introduced into *P. larvae* DTK384 by electroporation as described above. Transformants carrying pJ18TS1mac were selected on MYPGP agar containing 1 μg/ml of TS and a representative transformant (DTK384pJ18TS1mac) was used for further analyses (Table 2).

### Antimicrobial Susceptibility Tests for Putative Macrolide Resistance Gene Expression Vector/pJ18TS1mac- Introduced *P. larvae*

Putative macrolide resistance gene expression vector-introduced *P. larvae* DTK384 and pMX2-TA-introduced DTK384 (DTK384control) were cultured on MYPGP agar supplemented with 16 μg/ml of CP for two days under air plus 5% CO_2_ conditions. The pJ18TS1mac-introduced DTK384 (DTK384pJ18TS1mac) was cultured on MYPGP agar supplemented with 2 μg/ml of TS under the same conditions. Antimicrobial susceptibility tests of the strains for TS, erythromycin (EM; FUJIFILM Wako Pure Chemical Corp.) and LCM were performed by agar dilution methods according to CLSI, M07-A10, except that MICs were measured on MYPGP agar plates after a 48-h incubation at 35°C under air plus 5% CO_2_ conditions. The antimicrobial concentration range tested was 0.0031–256 μg/ml. *S. aureus* ATCC 29213 and *Enterococcus faecalis* ATCC 29212 cultured on MH agar for 20 h at 37°C under aerobic and air plus 5% CO_2_ conditions, respectively, were employed as the quality control strains, and antimicrobials used were confirmed to have acceptable potency after a 20-h incubation of *E. faecalis*- and *S. aureus*-inoculated MH agar plates at 35°C under aerobic conditions.

### pJ18TS1mac Stability Tests in *P. larvae*

To investigate pJ18TS1mac stability in *P. larvae* under non-selective (no antimicrobial) conditions, DTK384pJ18TS1mac cultured for approximately two days on MYPGP agar containing 2 μg/ml of TS at 35°C under air plus 5% CO_2_ conditions was suspended in MYPGP broth to an optical density at 600 nm of approximately 0.2, and 10 μl of the suspension (inocula) was inoculated into 2 ml of non-selective MYPGP broth. After an approximately 24-h culture at 35°C with shaking (200 rpm), 1–2 μl of the culture was inoculated into 2 ml of the fresh non-selective MYPGP broth and further cultured for 24 h under the same conditions. After 5, 10, 15 and 25 cycles of the non-selective broth culture steps, serial dilutions of the culture were inoculated onto selective (2 μg/ml of TS) or non-selective MYPGP agar plates. After 2–3-day culture of the plates at 35°C under air plus 5% CO_2_ conditions, plasmid retention rates were calculated by the following formula: the plasmid retention rate = the number of colonies grown on selective MYPGP agar plate/the number of colonies grown on non-selective MYPGP agar plate × 100%. Plasmid retention rates of inocula were also measured similarly. Data were collected from five independent culture tubes. Differences in the average retention rate of pJ18TS1mac among inocula and bacteria at 5, 10, 15, and 25 passages were analyzed by Friedman’s test. All tests were performed using EZR (Saitama Medical Center, Jichi Medical University, Saitama, Japan) (Kanda, 2013), which is a graphical user interface for R (The R Foundation for Statistical Computing, Vienna, Austria). A value of *P* < 0.05 was considered as the threshold for significance.

## Results

### Bacteria Isolated From Japanese Honey and Their Antimicrobial Susceptibility

**FIGURE 1:**
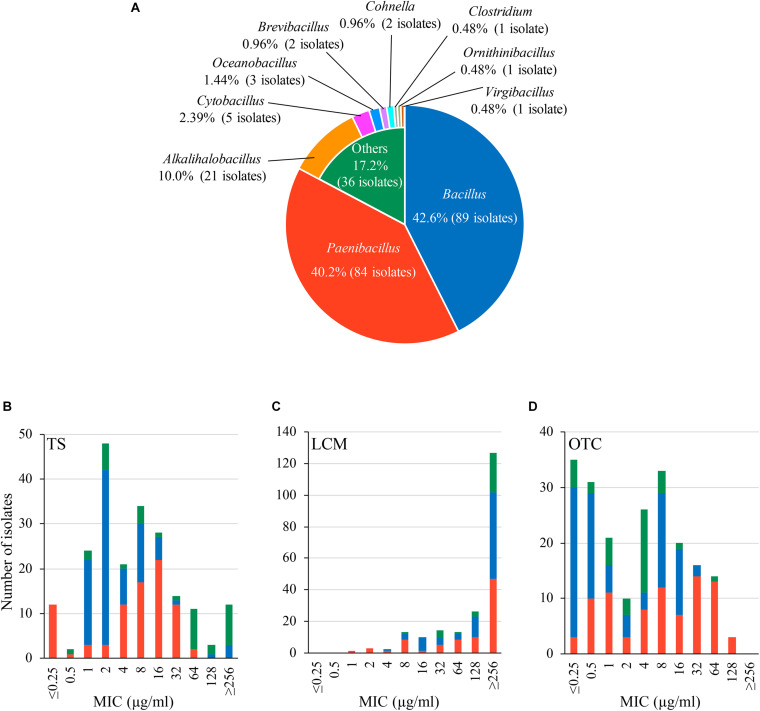
Characteristics of bacteria isolated from Japanese honey on 1 μg/ml-tylosin (TS)-containing agar media. **(A)** Proportion of genera found in the bacterial isolates. Genera/species of the isolates was identified by 16S rRNA gene sequencing. **(B–D)** Susceptibility of the isolates to TS, lincomycin (LCM) and oxytetracycline (OTC), respectively. Minimum inhibitory concentrations (MICs) of TS, LCM and OTC were measured by broth microdilution/agar dilution methods. The proportion of isolates with different genera in each MIC is indicated by different colors according to **(A)**.

In this study, we obtained 209 bacterial isolates from 48 of the 53 (90.6%) Japanese honey samples under 1 μg/ml-TS conditions (Supplementary Table 2). As of September 2020, the 209 isolates were classified into 10 genera by 16S rRNA gene sequencing, and at least 52 species existed in the isolates (Figure 1A and Supplementary Table 2). *Bacillus* (89 isolates, 42.6%) and *Paenibacillus* (84 isolates, 40.2%) were the two most common genera found in the isolates, followed by *Alkalihalobacillus* (21 isolates, 10.0%), *Cytobacillus* (5 isolates, 2.39%), *Oceanobacillus* (3 isolates, 1.44%), *Brevibacillus* (2 isolates, 0.96%) and *Cohnella* (2 isolates, 0.96%) (Figure 1A). *Clostridium*, *Ornithinibacillus* and *Virgibacillus* were also found, but only in one isolate each (Figure 1A). All isolates were Gram-positive spore-forming bacteria. Except for the anaerobic bacterium *Clostridium beijerinckii* J33TS5, all isolates were aerobic or facultatively anaerobic. At the species level, *Bacillus licheniformis*, *Paenibacillus cineris*, *Alkalihalobacillus clausii* and *Paenibacillus dendritiformis* were frequently isolated, and found in 45.3, 35.8, 30.2, and 26.4% of honey samples, respectively. Under the 1 μg/ml-TS conditions, neither *P. larvae* nor *M. plutonius* was isolated from the Japanese honey. *Clostridium botulinum*, cereulide synthetase gene-positive *Bacillus cereus* and *Bacillus anthracis*, which may cause public health problems, were also not isolated in this study.

Results of the antimicrobial susceptibility tests are shown in Figures 1B–D and Supplementary Table 2. In addition to TS, LCM and OTC are used for controlling AFB in other countries (Reybroeck et al., 2012; Krongdang et al., 2017). Although LCM and OTC are not approved drugs in Japan, the use of these drugs may be considered in the future if TS-resistant *P. larvae* will spread in Japan. Therefore, we also employed LCM and OTC for the tests. MICs of the antimicrobials for the honey-derived isolates were widely distributed, and the MIC range of TS, LCM and OTC was ≤ 0.25– ≥ 256, 1– ≥ 256 and ≤ 0.25–128 μg/ml, respectively. Although bacteria in honey were screened under 1 μg/ml-TS conditions, MICs of TS were 1 μg/ml or lower in some isolates. However, most (94.3%) of the isolates exhibited lower susceptibility to TS than Japanese *P. larvae* strains (MIC of TS, 0.125–0.25 μg/ml) (Ueno et al., 2018), and 12 isolates had a MIC of 256 μg/ml or higher (Figure 1B and Supplementary Table 2). Most of the isolates also demonstrated low susceptibility (MIC50, ≥ 256 μg/ml) to LCM, which is structurally different but functionally similar to macrolides (Figure 1C and Supplementary Table 2). Moreover, in 122 (58.4%) isolates, MICs of OTC were higher than those for Japanese *P. larvae* strains (MIC, ≤ 1 μg/ml) (Ueno et al., 2018) (Figure 1D and Supplementary Table 2). This suggests a wide distribution of antimicrobial resistance genes in honey-derived bacteria.

### Antimicrobial Resistance Genes in Honey-Derived Bacteria Detected by Genome Sequence Analysis

In order to identify genes conferring antimicrobial resistant phenotypes of the honey-derived isolates, we selected 50 isolates for genome sequencing (Table 1). The isolates were selected from various species. When there were multiple isolates in one species, 1–3 isolate(s), which demonstrated low susceptibility to TS and/or LCM, were selected as representatives. The summary of the genome sequencing results is shown in Supplementary Table 4 in Supplementary Materials. The sizes of the assembled genome of the isolates ranged from 3.48 to 7.44 Mbp, and there were 3,463–6,685 predicted coding sequences (Supplementary Table 4). RGI detected 379 macrolide, lincosamide and/or tetracycline resistance genes from the 50 isolates, and 59 met the perfect or strict criteria of the analysis^3^ (Supplementary Table 5 in Supplementary Materials). Among the resistance genes, 156, 166 and 207 genes, including 28, 46 and 11 perfect or strict hit genes, were predicted to confer macrolide, lincosamide and tetracycline resistance, respectively (Table 1 and Supplementary Table 5).

**FIGURE 2:**
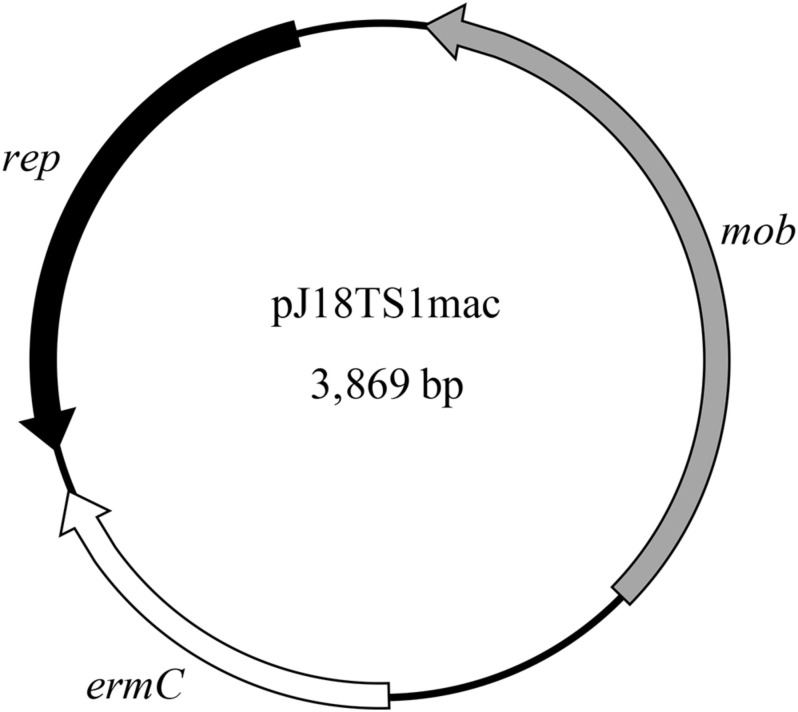
Map of pJ18TS1mac. *rep*, replication protein gene; *mob*, mobilization protein gene; *ermC*, 23S rRNA methyltransferase gene (macrolide resistance gene).

**FIGURE 3:**
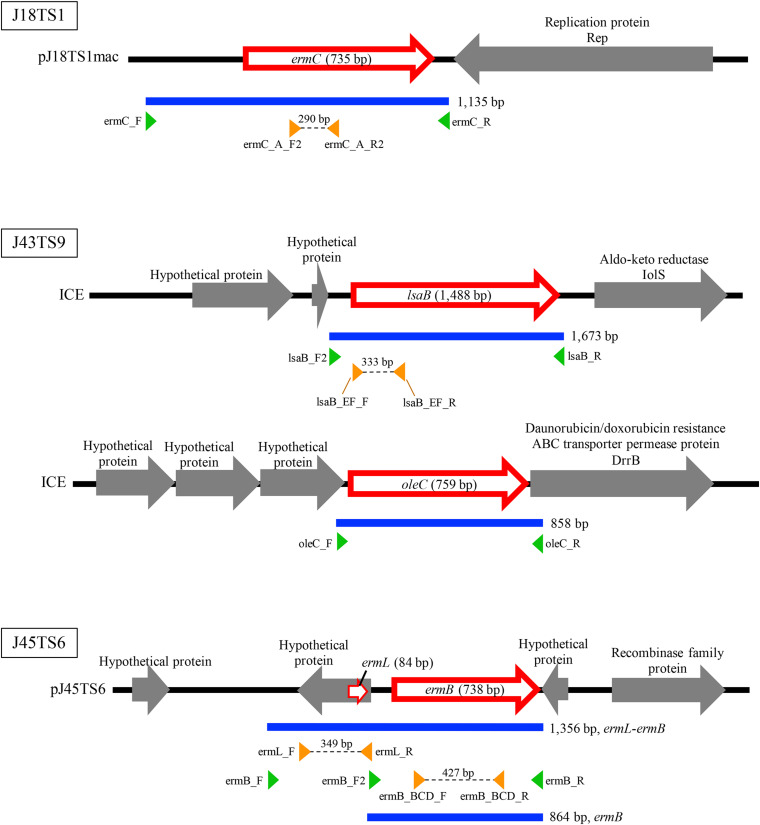
Schematic representation of macrolide resistance gene regions found in putative mobile genetic elements of honey-derived isolates. Blue lines indicate regions amplified by PCR for construction of antimicrobial resistance gene expression vectors, and green arrowheads represent primers used for PCR. Orange arrowheads represent primers used to confirm expression of the introduced macrolide resistance genes in *P. larvae*.

PlasmidFinder and additional sequence analysis identified three plasmids carrying antimicrobial resistance genes (Table 1). Two of the three plasmids (pJ18TS1mac and pJ18TS1tet), which carry the macrolide resistance gene *ermC* and tetracycline resistance gene *tetL*, respectively, were found in *Oceanobacillus oncorhynchi* subsp. *incaldanensis* isolate J18TS1. Both plasmids were relatively small in size (less than 4.7 kbp) and possessed the *mob* gene (Figure 2, accession number LC586958 [pJ18TS1mac] and LC596398 [pJ18TS1tet]), suggesting that they are mobilizable plasmids. Another plasmid pJ45TS6 found in *Paenibacillus* sp. isolate J45TS6 was 51.4 kbp in size, and carried the macrolide resistance gene *ermB* and tetracycline resistance gene *tetL*. Although not detected by RGI, *ermL*, which was predicted to control the expression of *ermB*, was identified in the upstream region of the *ermB* gene by Blast analysis^8^ (Figure 3). In addition, two putative macrolide resistance genes, *lsaB* and *oleC*, were identified in an ICE region of *Paenibacillus cineris* isolate J43TS9 (Table 1). In contrast, no intact prophages with antimicrobial resistance genes were identified in this study.

### The *ermC* and *ermL-ermB* Regions Possessed by Plasmids pJ18TS1mac and pJ45TS6, Respectively, Confer Macrolide and Lincosamide Resistance to *P. larvae*

**FIGURE 4:**
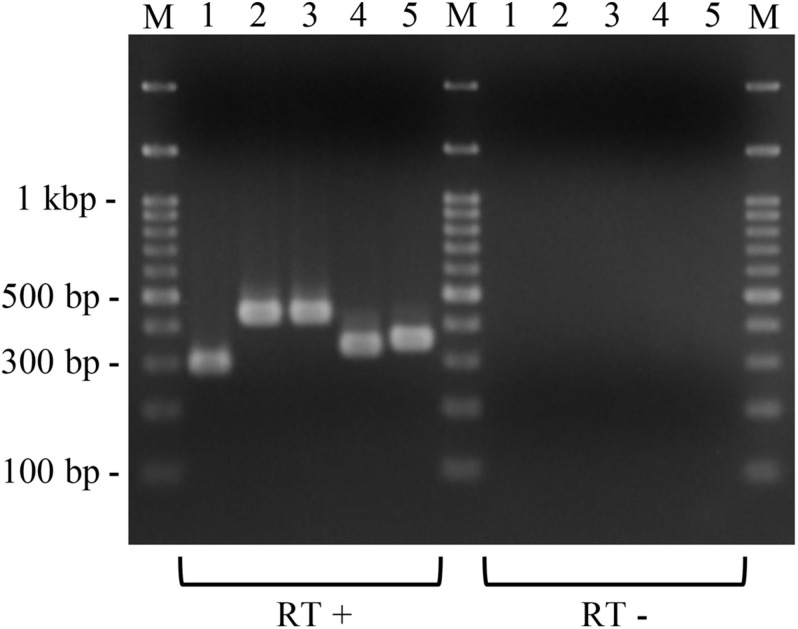
Expression of the introduced putative macrolide resistance genes in *P. larvae*. Total RNA was extracted from *P. larvae* strains cultured on MYPGP agar containing 16 μg/ml of chloramphenicol, and the expression of macrolide resistance genes was assessed by reverse transcription (RT)-PCR. To exclude DNA contamination in the RNA samples, total RNA, which was not treated with reverse transcriptase (RT -), was also used as the template for PCR. Lane 1: *ermC* expression in DTK384ermC; lane 2: *ermB* expression in DTL384ermLB; lane 3: *ermB* expression in DTK384ermB; lane 4: *lsaB* expression in DTK384lsaB; lane 5: *ermL* expression in DTL384ermLB. All introduced putative macrolide resistance genes were expressed in *P. larvae*.

To investigate whether the putative macrolide resistance genes found in MGEs (Figure 3 and Table 1) have the potential to confer macrolide resistance to *P. larvae*, we introduced them into *P. larvae* using pMX2-TA and assessed antimicrobial susceptibility of the transformants. As isolation of ERIC II strains from AFB cases is recently increasing in Japan (Ueno et al., 2018), an ERIC II strain, DTK384, was used as a representative host for the analysis. Among the five target resistance genes/region (blue lines in Figure 3), the *oleC* gene of isolate J43TS9, which encodes ATP-binding cassette (ABC) antibiotic efflux pump, was unable to be cloned into pMX2-TA in *E. coli*, possibly due to toxicity of the product to *E. coli*; therefore, this gene was not used for further analysis. As shown in Figure 4, the *ermC*, *ermL-ermB, ermB* and *lsaB* genes were introduced into and expressed in *P. larvae* DTK384, and the resultant *P. larvae* strains were designated DTK384ermC, DTK384ermLB, DTK384ermB and DTK384lsaB, respectively (Table 2). As a control, DTK384 with the empty vector pMX2-TA (DTK384control) was also prepared. In addition to the 16-membered ring macrolide TS, EM was used as a representative 14-membered ring macrolide for antimicrobial susceptibility tests. As macrolide resistance genes may confer cross-resistance to lincosamides, LCM was also used for the tests.

As expected, the antimicrobial susceptibility of DTK384control (MICs of TS, EM and LCM; 0.25, 0.0625, and 0.25 μg/ml, respectively) was almost the same as that of the parent strain DTK384 (MICs of TS, EM and LCM; 0.25, 0.125, and 0.25 μg/ml, respectively) (Table 2). In contrast, introduction of *ermC* into *P. larvae* DTK384 significantly reduced the susceptibility of the strain (DTK384ermC) to all the three antimicrobials tested (MICs of TS, EM and LCM; 8, > 256 and > 256 μg/ml, respectively) (Table 2). Introduction of *ermB* into DTK384 also significantly reduced the susceptibility of the strain (DTK384ermB) to EM and LCM (MICs; 64 and > 256 μg/ml, respectively). In addition, co-introduction of *ermL* and *ermB* into the strain made *P. larvae* (DTK384ermLB) more resistant to EM (MIC, > 256 μg/ml) than DTK384ermB. However, DTK384ermLB was still relatively highly susceptible to TS (MIC, 1 μg/ml) even in the presence of both *ermL* and *ermB* (Table 2). On the other hand, *lsaB* did not confer macrolide resistance to *P. larvae* (MICs of TS, EM and LCM for DTK384lsaB; 0.25, 0.125, and 0.25 μg/ml, respectively) (Table 2). Thus, among the genes tested, the *ermC* genes in pJ18TS1mac, and *ermL* and *ermB* genes in pJ45TS6 have the potential to confer macrolide and lincosamide resistance to *P. larvae*.

### The Macrolide-Resistant Plasmid pJ18TS1mac Can Be Stably Inherited in the *P. larvae* Population

As J18TS1 possesses putative ICEs, which encode type IV secretion system components, in the chromosome (Supplementary Table 6 in Supplementary Materials), pJ18TS1mac carrying the *mob* gene (Figure 2) may be horizontally transferable to other bacterial strains/species using the type IV secretion systems. Isolate J45TS6 also possesses putative ICEs in the chromosome (Supplementary Table 6). Although pJ45TS6 was not considered to be a transferable plasmid due to the absence of the *mob* gene, we performed filter mating tests using both J18TS1 and J45TS6 as donors and *P. larvae* M20 as the recipient in order to investigate whether the isolates can directly transfer their macrolide-resistant plasmids to *P. larvae*. However, neither J18TS1 nor J45TS6 transferred the plasmids to *P. larvae* under the experimental conditions used in this study.

However, we cannot exclude the possibility that the macrolide-resistant plasmids, in particular pJ18TS1mac, will be horizontally transferred to *P. larvae* under natural environmental conditions. If transferred plasmids are stably maintained in *P. larvae*, TS-resistant *P. larvae* may spread in Japan like OTC-resistant strains in other countries. Therefore, in order to investigate whether pJ18TS1mac can replicate in and be stably inherited by *P. larvae*, we introduced the plasmid into *P. larvae* DTK384 by electroporation and investigated plasmid retention rates in the strain under non-selective culture conditions.

**FIGURE 5:**
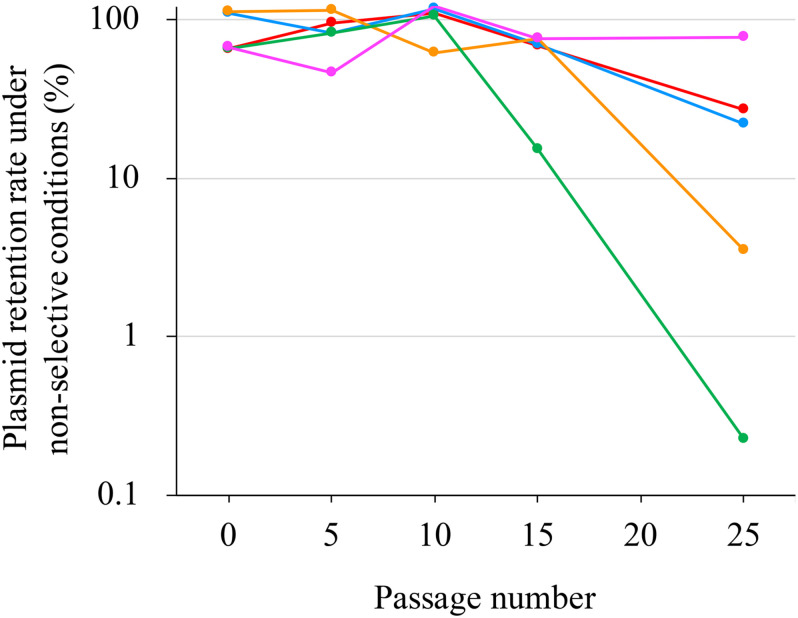
Stability of pJ18TS1mac in *P. larvae*. pJ18TS1mac-introduced DTK384 (DTK384 pJ18TS1mac) was cultured at 35°C in MYPGP broth under non-selective conditions, and bacteria were subcultured every 24 h by inoculating 1–2 μl of the culture into a fresh non-selective MYPGP broth. After 5, 10, 15 and 25 cycles of the culturing steps, serial dilutions of the culture were inoculated onto selective (2 μg/ml of TS) and non-selective MYPGP agar plates, and plasmid retention rates were calculated by the following formula: the plasmid retention rate = the number of colonies grown on selective MYPGP plate/the number of colonies grown on non-selective MYPGP plate × 100%. Plasmid retention rates of inocula for the first culture step were also measured. Data was collected from five independent culture tubes and results of each culture tube are shown in different colors.

pJ18TS1mac was introduced into *P. larvae* DTK384, and the strain (DTK384 pJ18TS1mac) became resistant to macrolide and lincosamide antibiotics as expected (MICs of TS, EM and LCM for DTK384pJ18TS1mac: 16, > 256 and > 256 μg/ml, respectively) (Table 2). Even after 10 passages under non-selective (no antibiotic) culture conditions, almost all *P. larvae* cells stably retained the plasmid. After 15 passages, the average plasmid retention rate decreased to 61.11 ± 23.2%, but the rate remained approximately 70% in four of the five test culture tubes. Although the average retention rate decreased to 26.12 ± 27.80% after 25 passages, the rate varied depending on the culture tubes (0.22–77.78%) and there were no significant differences in the average retention rate throughout the experimental period (Friedman’s test, *P* = 0.0755) (Table 3 and Figure 5). This suggested that the macrolide resistance plasmid can be inherited in the *P. larvae* population for a long period even under non-selective conditions after acquiring the plasmid.

**TABLE 3:** Stability of pJ18TS1mac in *P. larvae* under non-selective conditions^a^.

Passage number under non-selective conditions	Average plasmid retention rate ± standard deviation (%)^b^
0 (inocula)	84.0 ± 22.25
5	84.15 ± 22.51
10	103.51 ± 21.87
15	61.11 ± 23.2
25	26.12 ± 27.8

*^a^DTK384pJ18TS1mac (described in Table 2) was used for plasmid stability tests. ^b^Data were collected from five independent culture tubes. There were no significant differences in the average retention rate throughout the experimental period (Friedman’s test, P = 0.0755).*

## Discussion

OTC and tetracycline-resistant *P. larvae* strains have been isolated in several countries. The resistant strains were suggested to develop, at least in part, by horizontal gene transfer of tetracycline resistance genes via MGEs (Evans, 2003; Murray and Aronstein, 2006; Alippi et al., 2007, 2014; Murray et al., 2007; Krongdang et al., 2017), and bacteria in honey may be a source of the antimicrobial resistance genes (López et al., 2008). Therefore, elucidation of the resistome in honey is important to predict the future evolution of antimicrobial resistance in foulbrood pathogens. In this study, we isolated bacteria from Japanese honey for the resistome analysis and found macrolide resistance genes on MGEs, which may confer TS-resistance if transmitted to *P. larvae*.

In the recent 16S rRNA gene amplicon sequencing for investigating microbiota in honey, *Lactobacillus kunkeei* (*Apilactobacillus kunkeei*), *Parasacharribacter apium* (*Bombella apis*), *Fructobacillus fructosus*, *Gilliamella apicola*, Xanthomonadales and Actinomycetales were detected as major bacterial species/groups at the DNA level (Floyd et al., 2020). Other previous studies reported that aerobic spore-forming bacteria of the genera *Paenibacillus* and *Bacillus* are commonly found in honey (Snowdon and Cliver, 1996; Alippi et al., 2004; López and Alippi, 2007; López et al., 2008). In this study, *Paenibacillus* and *Bacillus* were also the two major genera found in the isolates from Japanese honey, and all isolates from the Japanese honey were spore-forming bacteria. Although it is unknown why major bacterial species/groups detected by 16S rRNA gene amplicon sequencing (Floyd et al., 2020) were not isolated in this study, the use of 1 μg/ml-TS-containing media at the first bacterial isolation step and the antimicrobial characteristics of honey itself may have affected the repertoire of live bacteria recovered by the culture methods. Alternatively, some honey samples used in this study may have been heated after harvesting and the heating step may have affected non-spore forming bacteria in the honey such as *A. kunkeei*, *B. apis* and *F. fructosus*.

In this study, *P. larvae* and *M. plutonius* were not isolated from Japanese honey under the 1 μg/ml-TS conditions. This suggests that Japan is still free from TS-resistant foulbrood pathogens. However, we cannot rule out the possibility that we have missed TS-resistant foulbrood pathogens in honey samples. For isolation of foulbrood pathogens from samples with other bacteria, it is necessary to use selective media supplemented with appropriate antimicrobials such as nalidixic acid and pipemidic acid (de Graaf et al., 2013; Forsgren et al., 2013). In this study, we did not use such selective media, and this may have caused overgrowth of other honey-derived bacteria, making the recovery of the pathogens difficult. In addition, culture methods are known to not be effective for detecting foulbrood pathogens from honey (Nordström et al., 2002; Gillard et al., 2008; Forsgren et al., 2013; Bassi et al., 2018). Therefore, further investigation using selective media and/or molecular techniques is needed to clarify the contamination status of *P. larvae* and *M. plutonius* in Japanese honey.

Although TS-containing media was used, some honey-derived isolates were highly susceptible to TS. In particular, the TS susceptibility of 12 isolates was at a similar level to that of Japanese *P. larvae* strains (i.e., MIC of TS, ≤ 0.25 μg/ml). As suggested by previous studies (Snowdon and Cliver, 1996; Alippi et al., 2004; López and Alippi, 2007; López et al., 2008), the presence of spore-forming bacteria in honey was expected. Therefore, in order to germinate as many spores as possible, we cultured honey sediment-inoculated agar media for 2–4 days at the first isolation step. During the relatively long cultivation step, the potency of TS may have decreased gradually, and TS-susceptible bacteria may have grown on the media. Alternatively, because of efficient TS inactivation by macrolide-resistant bacteria coexisting in the same honey, the antibiotic may not be able to inhibit the growth of these TS-susceptible bacteria. Indeed, our genome analysis suggested the presence of bacteria in honey, which produce enzymes involved in macrolide inactivation (macrolide phosphotransferases) (Supplementary Table 5). However, such highly TS-susceptible bacteria were the minority among the honey-derived isolates retrieved in this study. MICs of TS for the honey-derived isolates varied (≤ 0.25– ≥ 256 μg/ml), and the majority of the isolates exhibited lower TS susceptibility than Japanese *P. larvae* strains (Ueno et al., 2018). Although breakpoints for antimicrobials tested have not yet been established for the bacterial species identified in this study, some isolates were highly resistant to TS (Figure 1 and Supplementary Table 2). These suggested the presence of multiple macrolide resistance mechanisms in honey-derived bacteria. Indeed, a variety of putative macrolide resistance genes involved in macrolide efflux, inactivation, target alteration and target protection were found in the representative isolates analyzed by genome sequencing (Supplementary Table 5).

In addition to macrolide resistance genes, a variety of lincosamide and tetracycline resistance genes were also detected in the genomes of the representative isolates by RGI (Supplementary Table 5). Consistent with this, many isolates from honey exhibited low susceptibility to LCM and OTC. In particular, 60.8% of the isolates were highly resistant to LCM (MICs, ≥ 256 μg/ml). Although the details were not reported in this study, RGI also detected many putative resistance genes for other drug classes, including aminoglycoside, phenicol, fluoroquinolone, glycopeptide and β-lactam antibiotics, in the genome of the honey-derived isolates (an example of RGI analysis results is shown in Supplementary Table 7 in the Supplementary Materials). This suggests the presence of many (and probably multidrug) resistant bacteria in honey and support the previous hypothesis that bacteria in honey are a reservoir of resistance genes (López et al., 2008).

Macrolides and lincosamides are antibiotics that inhibit protein synthesis, particularly the elongation step, by targeting the bacterial ribosome. In the ribosome, the peptidyl-transferase center (PTC) and adjacent nascent peptide exit tunnel (NPET) are the key players in protein elongation and function in catalyzing the peptide bond formation and the emergence of the nascent chain, respectively. Macrolides bind to a site within the NPET, whereas lincosamides are PTC-targeting antibiotics (Schlünzen et al., 2001; Wilson, 2009; Dunkle et al., 2010; Kannan and Mankin, 2011). Among the four genes tested, *lsaB* located in an ICE of *P. cineris* J43TS9 is considered to encode an ATP-binding cassette F (ABC-F) type ribosomal protection protein. ABC-F proteins are known to confer resistance to a number of clinically relevant antibiotics targeting the ribosome PTC/NPET region by interacting with the ribosome and displacing the bound drug (i.e., ribosomal protection mechanism) (Kerr et al., 2005; Reynolds and Cove, 2005; Sharkey et al., 2016). However, expression of *lsaB* in *P. larvae* affected neither macrolide nor lincosamide susceptibility (Table 2). ABC-F proteins have been classified into three groups based on antibiotic resistance (Msr homologs [macrolides and streptogramin B], Vga/Lsa/Sal homologs [lincosamides, pleuromutilins and streptogramin A] and OptrA homologs [phenicols and oxazolidinones]) (Sharkey et al., 2016; Su et al., 2018). Although the gene of J43TS9 was detected as *lsaB* by RGI, its product was only loosely similar to the reference sequence of LsaB in CARD (Table 2 and Supplementary Table 5). Based on this relatively low similarity to LsaB and our results, neither macrolides nor lincosamides are the targets of the *lsaB* product of J43TS9.

In contrast, introduction of the *ermC* and *ermB* genes located in plasmids of J18TS1 and J45TS6, respectively, into *P. larvae* DTK384 markedly reduced the susceptibility of the strain to EM and LCM (Table 2). This was not surprising because the putative products of the two genes were similar to the methyltransferases that modify a specific adenine residue (A2058, *E. coli* numbering) of 23S rRNA and confer macrolide-lincosamide-streptogramin B (MLS_B_) resistance (Franceschi et al., 2004; Weisblum, 1995). In the upstream region of *ermB*, an open reading frame (*ermL*) encoding the leader peptide was present, suggesting that expression of *ermB* is inducible. Leader peptide and inducible *erm* genes are known to constitute an operon, and expression of the *erm* gene is normally repressed due to sequestration of the *erm* ribosome binding site in the mRNA secondary structure. In the presence of macrolides, ribosomes stall during the translation of the leader peptide. The stall triggers a conformational rearrangement in mRNA, resulting in the opening of the *erm* ribosome binding site and activation of *erm* translation (Gaynor and Mankin, 2003). As expected from this, introduction of *ermL* further reduced the EM susceptibility of *P. larvae* (MIC of EM increased from 64 μg/ml to > 256 μg/ml); however, the 16-membered macrolide TS was still relatively effective against *P. larvae* (MIC, 1 μg/ml) (Table 2). Most *erm* genes are known to be induced by the 14- or 15-membered macrolides and not by 16-membered macrolides (Fyfe et al., 2016); therefore, in *P. larvae*, expression of the *ermB* gene may not have been efficiently induced by TS. Of note, the hypothetical protein gene located in the *ermL* region was also introduced into *P. larvae* in this study (Figure 3); however, antimicrobial susceptibility of DTK384ermLB suggests that the gene does not have the ability to greatly reduce the TS susceptibility of *P. larvae*.

In contrast to *ermB* in pJ45TS6, no leader peptide gene was found in the upstream region of the *ermC* gene in pJ18TS1mac, suggesting constitutive *ermC* expression in bacteria. Indeed, the MIC of TS for *P. larvae* DTK384 increased 32-fold only by introducing the *ermC* gene (DTK384ermC in Table 2). However, TS was still more effective in DTK384ermC (MIC, 8 μg/ml) than EM (> 256 μg/ml). Macrolides consist of a macrocyclic lactone ring carrying one or more sugar moieties (Gaynor and Mankin, 2003). EM carries two monosaccharides, cladinose and desosamine, at the C3 and C5 positions of the ring, respectively. When it binds to the 50S subunit of ribosomes, the macrocyclic lactone ring associates the NPET wall formed by positions 2057–2059 of 23S rRNA, whereas the monosaccharide moiety at the C5 position points toward the PTC. However, it cannot span the distance to the catalytic center (Hansen et al., 2002; Franceschi et al., 2004). In contrast, TS has a disaccharide at the C5 position of the ring and reaches further toward to the PTC (Hansen et al., 2002; Franceschi et al., 2004). Such structural features of TS may have increased the affinity of the drug with ribosomes even in the presence of methylated A2058 in 23S rRNA and exhibited stronger antimicrobial effects against *P. larvae* than EM.

However, proliferation of *ermC*-positive *P. larvae* in larvae will hardly be arrested even by TS. In the previous study by Reynaldi et al. (2017), beehives with 25,000 adult bees were treated with TS by dusting methods; i.e., the mixture consisting of 114 g of confectioner’s sugar, 5 g of cherry jelly and 1 g of TS was divided into four, 250 mg of TS to 30-g portions, and each portion was sprinkled over the ends of the hives’ top bars for four weeks at one-week intervals. According to the article, the mean concentration of TS in 2-day-old larvae measured by microbiological assay with *Geobacillus stearothermophilus* ATCC 12980 was always less than 4 μg/ml (Reynaldi et al., 2017). In another previous study, honey bee colonies containing around 30,000 worker bees were fed 500 g of synthetic honey with 200 mg or 400 mg of TS for 48 h, and the maximum concentration of TS in 48-h-old larvae was less than 1 μg/g even when treated with 400 mg of TS (Bernal et al., 2011). Similar to the study by Reynaldi et al. (2017), TS is used by dusting methods in Japan, and only 150 mg of TS is used for 10,000 adult bees. The dose of TS in Japan is lower than and almost equivalent to those in the studies by Reynaldi et al. (2017) and Bernal et al. (2011), respectively; therefore, the TS concentration in young larvae is expected to be less than 1–4 μg/ml. In contrast, MICs of TS for DTK384ermC and DTK384pJ18TS1mac were 8–16 μg/ml, suggesting that *ermC*-positive *P. larvae* is clinically resistant to TS. Therefore, acquisition of *ermC* by *P. larvae* is one of the greatest threats to apiculture in Japan.

As the *ermC*-positive plasmid pJ18TS1mac possesses the *mob* gene (Figure 2), it is considered a mobilizable plasmid and may transfer to other bacterial cells using conjugation machineries encoded by other MGEs. Although pJ18TS1mac in *O. oncorhynchi* subsp. *incaldanensis* J18TS1 did not transfer to *P. larvae* DTK384 under the mating conditions tested in this study, we cannot exclude the possibility that the plasmid transfers to *P. larvae* under specific conditions in the natural environment. In Bergey’s manual (Heyrman and Vos, 2015), *O. oncorhynchi* subsp. *incaldanensis* is described to be susceptible to EM and LCM; however, isolate J18TS1 was resistant to TS and LCM, and *ermC* in pJ18TS1mac was able to confer EM resistance. Therefore, J18TS1 is also considered to have acquired pJ18TS1mac from other bacteria horizontally, and thus, it is also possible that *P. larvae* acquires pJ18TS1mac or similar plasmids from other bacteria in the natural environment. As demonstrated in this study, pJ18TS1mac can be maintained in the *P. larvae* population for a long period. Stable plasmid inheritance can be achieved by several means such as (i) integration into the host chromosome, (ii) incorporating genes that participate in plasmid stable inheritance, like the toxin-antitoxin systems, into the plasmid, (iii) incorporating partitioning systems such as the ParABS systems and (iv) reducing plasmid size and increasing plasmid copy number in bacterial cells (Pluta and Espinosa, 2018). As pJ18TS1mac encodes a rolling circle replication initiator protein (Rep) (Figure 2; accession number LC586958), it is considered a rolling circle replicating-plasmid. Rolling circle replicating-plasmids usually do not carry specialized partitioning systems but ensure their stable inheritance because of their elevated plasmid copy numbers so that the probability of plasmid loss is reduced (Pluta and Espinosa, 2018). Indeed, pJ18TS1mac possesses neither the toxin-antitoxin systems nor the ParABS systems and its size is small (3,869 bp) (Figure 2). Therefore, pJ18TS1mac may have been maintained stably in *P. larvae* because of its high copy number in the cells. Due to the stable inheritance, once *P. larvae* acquires this plasmid in the field, TS-resistant *P. larvae* may be selected by the use of the prophylactic for AFB and spread throughout Japan. A putative mobilizable plasmid carrying the *tetL* gene (pJ18TS1tet) was also found in J18TS1. Although the use of OTC in apiculture is not allowed in Japan, OTC-resistant *P. larvae* may also be selected and spread in Japan if the drug is used illegally. Furthermore, the use of these drugs as prophylactics may affect the antimicrobial susceptibility of another foulbrood pathogen, *M. plutonius*, as seen by the use of MRM in Japan (Takamatsu et al., 2018). Although microorganisms in honey primarily originate from pollen, the digestive tracts of honey bees, dust, air, earth and nectar, honey can also be contaminated with microorganisms during and after harvesting process by humans, equipment and buildings (Snowdon and Cliver, 1996). Therefore, the isolates and their resistance genes found in commercially available honey in this study may not have necessarily existed in comb honey. However, our present study suggests the possibility of the future emergence of antimicrobial-resistant *P. larvae* by acquiring resistance genes from bacteria in honey. Therefore, it is necessary to use the prophylactic more carefully to prevent or delay the emergence. Development of novel molecular methods to detect resistance genes on MGEs together with foulbrood pathogens in honey will be helpful to use the prophylactic more carefully. Elucidation of the TS and LCM resistance mechanisms of North American *P. larvae* (Krongdang et al., 2017) is also required to predict and prevent the further emergence of antimicrobial resistant *P. larvae*.

## Data Availability Statement

The datasets presented in this study can be found in online repositories. The names of the repository/repositories and accession number(s) can be found in the article/Supplementary Material.

## Author Contributions

DT and MO designed the study and mainly performed the experiments. HK obtained genome sequence data, and MK analyzed the genome data. All authors contributed to the preparation of the manuscript and approved the final version.

## Conflict of Interest

The authors declare that the research was conducted in the absence of any commercial or financial relationships that could be construed as a potential conflict of interest.

## Footnotes

1 https://pubmlst.org/plarvae/

2 https://www.ezbiocloud.net

3 https://card.mcmaster.ca/analyze/rgi

4 https://github.com/arpcard/rgi

5 http://db-mml.sjtu.edu.cn/ICEberg/

6 http://phast.wishartlab.com

7 https://cge.cbs.dtu.dk//services/PlasmidFinder/

8 https://blast.ncbi.nlm.nih.gov/Blast.cgi
